# Non-Target Suppression Supports the Formation of Representational Prioritization Under High Working Memory Load

**DOI:** 10.3390/brainsci15060633

**Published:** 2025-06-12

**Authors:** Yaya Zhang, Gongao Li, Xuezhu Hu, Peng Zhang, Jinhong Ding

**Affiliations:** Learning and Cognition Key Laboratory of Beijing, School of Psychology, Capital Normal University, Beijing 100048, China; 15600056176@163.com (Y.Z.); 15081990917@163.com (G.L.);

**Keywords:** visual working memory, memory load, representational prioritization, target enhancement, non-target suppression

## Abstract

**Background:** Target enhancement and non-target suppression are two critical mechanisms underlying representational prioritization in visual working memory (VWM). However, it remains unclear how VWM load modulates these prioritization mechanisms. **Methods:** Using EEG combined with a retro-cue paradigm, this study investigated how representational prioritization emerges under low (Experiment 1) and high (Experiment 2) memory load conditions. **Methods:** Behavioral results showed that under low load, both target and non-target items benefited from retro-cue. ERP analyses revealed significantly larger P2 and P3b amplitudes in response to valid compared to neutral retro-cues, whereas no significant contralateral delay activity (CDA) component was observed. Under high load, cueing benefits were restricted to target items, whereas non-target items suffered impaired performance. ERP analyses again showed enhanced P2 and P3b amplitudes for valid compared to neutral retro-cues, but a significant CDA component was also observed. Time–frequency analyses further revealed frontal theta synchronization (ERS) and posterior alpha desynchronization (ERD) under both load conditions. Notably, theta–alpha phase–amplitude coupling (PAC) was significantly stronger for valid than neutral retro-cues under low load, whereas under high load, PAC did not significantly differ between cue conditions. **Conclusions:** Together, these findings suggest that target enhancement serves as a stable mechanism for representational prioritization, whereas non-target suppression critically depends on resource availability. VWM load systematically shapes representational prioritization through modulation of oscillatory timing characteristics and inter-regional neural coordination.

## 1. Introduction

Visual working memory (VWM) is a cognitive system that temporarily maintains and actively manipulates visual information relevant to the current task [1,2]. However, due to its limited resources, individuals are unable to simultaneously process all visual information held in VWM [3,4]. Therefore, internal attentional mechanisms are required to selectively regulate information processing. Specifically, individuals must prioritize task-relevant visual representations to efficiently allocate their limited cognitive resources. This selective processing, known as representational prioritization, is considered a key mechanism underlying complex visual cognition [5,6].

In recent years, the retro-cue paradigm has been widely used to investigate how internal attention dynamically regulates representational prioritization in VWM. This paradigm presents a cue between the end of encoding and the beginning of retrieval, directing attention to a specific visual memory item and thereby enhancing memory performance [7,8]. Two main mechanisms are considered to underlie this process: target enhancement and non-target inhibition. The target enhancement account proposes that retro-cues guide attentional resources toward the cued visual item, thereby strengthening its representational quality and accessibility in VWM [9,10]. In contrast, the non-target inhibition account emphasizes the active removal or suppression of uncued items, which reduces competition for limited resources and indirectly enhances the representation of the target [11,12]. An increasing body of evidence suggests that target enhancement and non-target inhibition are not mutually exclusive but rather can occur independently and are associated with distinct neural control mechanisms. Specifically, target enhancement is typically linked to frontal theta oscillatory activity, whereas non-target inhibition has been associated with alpha-band oscillations in occipital–parietal regions [13,14,15,16,17,18].

Although numerous studies have demonstrated that retro-cues can effectively modulate attentional allocation within VWM [19,20], there remains considerable debate over whether such effects are driven by target enhancement or non-target inhibition. For instance, imagine a student briefly glancing at a shopping list with several items (e.g., milk, bread, apples). After a moment, someone reminds the student to remember the apples. This functions like a valid retro-cue: the reminder helps the student focus their memory on the cued item (apples), thereby improving recall accuracy. Li et al. (2023) used a mixture modeling approach on behavioral data and found that retro-cues significantly increased the probability of target recall [21]. Moreover, multivariate pattern classification analysis based on EEG data revealed that approximately 500 ms after the presentation of a valid retro-cue, decoding accuracy for the target location rose significantly above chance and was positively correlated with individual behavioral benefits, providing support for the target enhancement account. However, decoding accuracy for non-target locations remained at chance throughout the retention interval, showing no signs of suppression. This absence of non-target inhibition may be attributed to the use of a low memory load in the experimental design. Additionally, although van Moorselaar et al. (2015) did not directly measure suppression or removal of non-targets, they observed that under high memory load, invalid retro-cues led to a marked decline in memory performance, and subsequent valid cues failed to fully restore performance [6]. Conversely, consider a situation where the student is initially told to remember apples, but later is unexpectedly asked whether bread was on the list. This would be like an invalid retro-cue, which diverts attention away from the target, making it harder to retrieve the originally prioritized information, especially when the list was long or complex. These behavioral patterns indirectly support the view that, under increased resource competition, non-target items may be weakened or even actively removed from VWM. While these findings offer important insights into target enhancement and non-target inhibition mechanisms, there remains a lack of direct comparisons of how target and non-target representations change under varying load conditions. Therefore, it remains unclear how visual working memory load influences the formation of representational prioritization.

In recent years, research on neural oscillations has provided critical insights into the mechanisms underlying the formation of representational prioritization in working memory. Frontal theta rhythms (4~8 Hz) are believed to play a central role in maintaining task goals and regulating prioritization, supporting the rhythmic allocation of attentional resources [17,22,23]. Meanwhile, alpha rhythms (8~12 Hz) in parietal–occipital regions are closely associated with the suppression of irrelevant perceptual input and the release of attention and may also reflect processes related to target enhancement and feature integration [24,25,26]. Recent evidence further suggests that long-range coordination between frontal and posterior brain regions plays a key role in retrospective attention. For example, Li et al. found that valid retro-cues significantly increased phase–amplitude coupling (PAC) between frontal theta phase and parietal alpha amplitude [21]. Importantly, the strength of this coupling predicted improved behavioral precision under valid cue conditions, as indicated by reduced guessing rates estimated using mixture modeling. These findings suggest that fronto-parietal PAC may serve as a fundamental mechanism supporting prioritized representations during the retention phase of working memory. Moreover, this long-range PAC differs from earlier reports of local theta–alpha coupling [27], supporting the view that retro-cues regulate the temporal dynamics of attention through distributed top-down control [23,28,29]. Given the flexible nature of working memory representations [30], such coupling may also support cognitive flexibility via dynamic reconfiguration of attentional priorities [31]. However, it remains unclear whether changes in memory load modulate theta synchronization, alpha desynchronization, and theta–alpha coupling, thereby influencing the relative contribution of target enhancement and non-target inhibition to representational prioritization.

To address the above research question, we employed a retro-cue task using real-world object images as stimuli [32,33,34]. By manipulating the number of memory items, we established two levels of VWM load: a low-load condition (Experiment 1) and a high-load condition (Experiment 2). We compared behavioral performance (memory accuracy) and neural responses (ERP components) to both target items (cued objects) and non-target items (uncued objects) across load conditions. To ensure cue validity (100%) while allowing for the assessment of responses to both target and non-target items, we used feature-based retro-cues (color cues) and introduced three types of probe stimuli (target, non-target, and novel items) under valid and neutral cue conditions. For example, if the retro-cue was “red,” the color of the probe stimulus was always red. A red item presented during encoding was treated as the target; 3an uncued item that changed to red at probe was treated as the non-target; and a red item that was not shown during encoding was treated as a novel item. If non-target suppression occurs, a retro-cue cost is expected for non-target items—namely, memory performance in the valid cue condition would be worse than in the neutral condition. If no non-target suppression occurs, no such cost should be observed.

In addition, previous ERP studies have identified several components associated with retro-cue effects in VWM tasks. The P2 component, typically observed over frontal–central electrodes around 150–250 ms post-cue, has been linked to attentional selection [35]. The P3b, a parietally distributed positivity peaking around 300–600 ms, is often interpreted as reflecting memory updating and resource allocation [36]. In addition, the contralateral delay activity (CDA) component reflects the number and resolution of items maintained in working memory and is sensitive to retro-cue reliability [33,34]. These findings provide important neural markers for distinguishing target enhancement and non-target suppression during the retention interval. Time–frequency analyses were further conducted to explore how VWM load modulates the neural oscillatory mechanisms underlying representational prioritization, focusing on frontal theta synchronization, parietal alpha desynchronization, and theta–alpha PAC between frontal and parietal regions.

## 2. Experiment 1

### 2.1. Materials and Methods

#### 2.1.1. Participants

Using G*Power 3.19 software [37], a power analysis was conducted with Cohen’s F = 0.25, α = 0.05, and power (1−β) ≈ 0.80, indicating a required sample size of 28 participants. To account for potential data loss and artifacts commonly encountered in EEG studies, 31 participants were initially recruited. Two participants were excluded due to excessive artifacts (i.e., fewer than 30 valid trials). The final sample included 29 participants (10 males), aged between 18 and 28 years. All participants were native Chinese of Han ethnicity, right-handed, with normal or corrected-to-normal vision, and reported no color blindness or color weakness. They voluntarily participated in the study and provided written informed consent prior to the experiment. Participants received monetary compensation after completing the experiment.

#### 2.1.2. Materials and Apparatus

All visual stimuli were selected from the standardized picture set by Snodgrass and Vanderwart [38]. Objects within each trial were matched based on their reported familiarity and visual complexity ratings to ensure comparability. Object colors included red, green, yellow, and blue, with RGB values of [255, 0, 0], [0, 255, 0], [0, 0, 255], and [255, 255, 0], respectively. Each object subtended a visual angle of 4 × 4°. During the encoding phase, two objects of different colors were simultaneously presented on a gray background (RGB: [192, 192, 192]), with a center-to-center distance of 7.5° between them.

EEG data were recorded using a 64-channel Neuroscan system with Ag/AgCl electrodes, following the extended 10/20 electrode placement system. Vertical electrooculogram (VEOG) was recorded from electrodes placed above and below the left eye, while horizontal electrooculogram (HEOG) was recorded from electrodes placed at the outer canthi of both eyes. Linked mastoids served as reference electrodes, and the ground electrode was located at the midpoint between FPz and Fz. EEG signals were sampled at 500 Hz, and electrode impedances were kept below 5 kΩ throughout recording.

The experimental procedure was programmed and executed using the open-source Psychopy 3.0 platform, running on a Windows 7 computer. Stimuli were displayed on a CRT monitor with a resolution of 1024 × 768 pixels and a refresh rate of 100 Hz. The viewing distance was approximately 60 cm. Participants completed the task while seated in a moderately lit, sound-attenuated, and electromagnetically shielded room and responded using a standard computer keyboard.

#### 2.1.3. Experimental Design and Procedure

The experiment adopted a 2-factor within-subjects design. The first independent variable was the type of retro-cue, which included two conditions: valid cue and neutral cue. The second independent variable was the type of probe stimulus, consisting of three conditions: target item, non-target item, and novel item. In the valid cue condition, to maintain cue validity, the probe item always matched the cue color. The probes were drawn from three sources: a memory item with the same color as the cue (target), a memory item with a different color transformed to the cue color (non-target), and a new item not shown during encoding (novel). To assess the role of retro-cues, the neutral cue condition included the same three types of probe stimuli for comparison.

**Figure 1:**
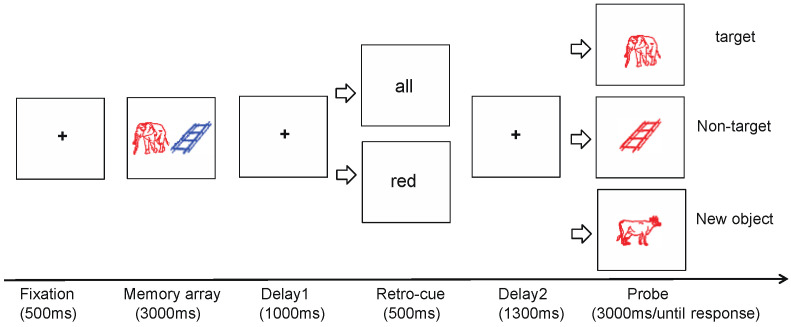
Experimental procedure of Experiment 1.

The trial structure of Experiment 1 is illustrated in Figure 1. Each trial began with a central fixation cross presented for 800~1000 ms. Next, a memory array consisting of two colored object images was displayed for 1000 ms, and participants were instructed to memorize both items. After another fixation cross for 1000 ms, a cue word (e.g., “red” for valid cue or “all” for neutral cue) appeared at the center of the screen for 500 ms. This was followed by another 1000 ms fixation period. Then, a single probe object appeared at the center of the screen, with the same size as the encoded items. Participants were instructed to judge whether the probe item was identical to one of the items in the memory array in both shape and color. If identical, participants pressed the “F” key; if not, they pressed the “J” key. The response window was 3000 ms. After the response, the next trial began.

A total of 300 trials were conducted in the experiment, with 150 trials for the valid cue condition and 150 trials for the neutral cue condition. Within each cue condition, the three probe types (target, non-target, and novel) were presented in 50 trials each. All trials were randomly divided into six blocks, each consisting of 50 trials. Prior to the formal experiment, participants completed at least 20 practice trials. A minimum 2 min break was provided between blocks to avoid fatigue.

#### 2.1.4. Data Analysis

**Behavioral Data** For Experiment 1, reaction times that deviated more than ±3 standard deviations from the mean were excluded, accounting for approximately 0.8% of all data.

**EEG Data** We primarily analyzed EEG data during the delay phase 2 under valid and neutral retro-cue conditions. Offline preprocessing was conducted using EEGLAB 14.1.2 toolbox based on MATLAB 2021b. EEG signals were re-referenced to the average of the left and right mastoids and filtered using a 0.1–40 Hz band-pass filter. Epochs ranging from −500 ms to 1800 ms relative to retro-cue onset were extracted. Independent Component Analysis (ICA) was applied to remove artifacts such as eye movements and muscle activity. Trials with amplitudes exceeding ±100 μV were excluded. Two participants were removed due to excessive artifacts (i.e., fewer than 30 valid trials). Baseline correction was applied using the −200 to 0 ms interval prior to cue onset. EEG waveforms were averaged for each condition. Based on grand-averaged waveforms and topographic maps, we defined regions and time windows of interest and used the mean amplitude within these windows for statistical analysis. The CDA component was assessed at electrodes P7/8 and PO7/8 by computing the difference waves between contralateral and ipsilateral sites relative to cue direction [7].

Time–frequency analysis EEG signals were segmented into −500 to 0 ms epochs relative to the retro-cue. To isolate non-phase-locked power, we first removed phase-locked ERP components to avoid contamination of induced oscillatory activity [39]. Instantaneous power and phase were estimated using short-time Fourier transform (STFT) with Hanning windows in the 2–30 Hz range (step size: 0.5 Hz). Power was baseline-corrected using the −500 to 0 ms interval and log-transformed. Power changes relative to baseline were referred to as ERD. This study primarily focused on theta-band activity (4–8 Hz), which is closely associated with the regulation of internal attention. Previous research has shown that frontal midline theta oscillations exhibit significant modulation following retro-cue presentation [17,18,21], though the exact timing of these effects remains inconsistent. Therefore, we estimated theta power at the frontal midline electrode Fz and selected the time windows based on the power peaks observed in the grand-averaged time–frequency plots. In addition, to investigate alpha-band activity (8–12 Hz) related to the redistribution of attentional resources, we referred to previous studies indicating that alpha power is predominantly distributed over posterior cortical areas [14,20]. Accordingly, alpha power was estimated across all parieto-occipital electrodes (P3/4, PO3/4, PO5/6, P7/8, PO7/8, O1/2, Pz, Oz).

PAC. To examine whether frontal theta phase modulates posterior alpha amplitude during retro-cue-driven attention, we performed PAC analysis. The Hilbert transform (hilbert.m) was applied to filtered EEG signals to extract theta phase and alpha amplitude. To ensure stability of PAC estimation, the analysis window was extended to cover the entire retention period (0~1800 ms, more than six theta cycles). Theta phase was extracted from the midfrontal electrode (Fz), and alpha amplitude from all scalp electrodes to assess frontoposterior connectivity. The modulation index (MI) was calculated to assess the degree of PAC [40]; a higher MI indicates stronger coupling between the two frequency bands.

Multivariate Pattern Analysis (MVPA). We further conducted time-resolved and cross-temporal decoding analyses using multivariate pattern analysis. A support vector machine (SVM) classifier with a linear kernel (regularization parameter = 1, stopping tolerance = 0.001) was applied. EEG data were downsampled to 50 Hz. For time-resolved decoding, trials were randomly shuffled and averaged in groups of five. Two-thirds of trials were used for training and one-third for testing, repeated 10 times. Undersampling was used to ensure equal trial numbers between conditions. Classification accuracies were averaged across all iterations for each participant. In cross-temporal decoding, classifiers trained at one time point were tested on all other time points to assess temporal generalization of neural representations. All MVPA analyses were performed using the NeuroRA toolbox [41]. To determine statistical significance, we used cluster-based permutation tests to compare decoding accuracy against chance level (50%). Specifically:(1) t-values were computed at each time point, and significant clusters were identified; (2) Cluster statistics were calculated as the sum of t-values within each cluster; (3) A total of 5000 permutations were conducted to create a null distribution of maximum cluster statistics; (4) *p*-values for each real cluster were obtained by comparing against the null distribution.

### 2.2. Results

#### 2.2.1. Accuracy and Response Time

**Figure 2:**
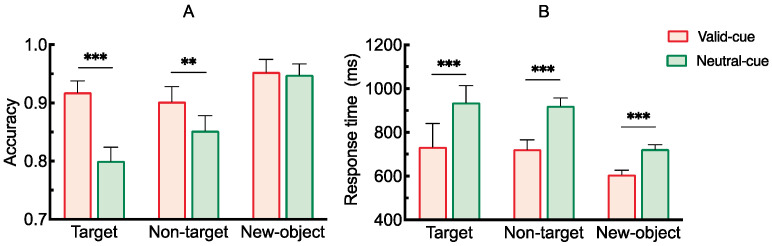
(**A**) Accuracy under different conditions, (**B**) Response time under different conditions (** *p* < 0.01; *** *p* < 0.001).

The results of the analysis of variance on accuracy under different conditions are shown in Figure 2A. The main effect of cue type was significant, *F*(1, 28) = 53.03, *p* < 0.001, *η*^2^_*p*_ = 0.65. The main effect of probe stimulus type was also significant, *F*(2, 56) = 10.38, *p* < 0.001, *η*^2^_*p*_ = 0.27. Moreover, there was a significant interaction between cue type and probe stimulus type, *F*(2, 56) = 22.81, *p* < 0.001, *η*^2^_*p*_ = 0.45. Post hoc comparisons revealed that when the probe stimulus was the target, accuracy under the valid cue condition was significantly higher than under the neutral cue condition [*t*(28) = 7.87, *p* < 0.001, Cohen’s *d* = 1.47]. When the probe stimulus was a non-target, accuracy was also significantly higher under the valid cue condition than under the neutral cue condition [*t*(28) = 3.77, *p* = 0.001, Cohen’s *d* = 0.49]. When the probe stimulus was a new object, the difference in accuracy between the two cue conditions was not significant [*t*(28) = 0.63, *p* = 0.539, Cohen’s *d* = 0.12].

The results of the analysis of variance on response time under different conditions are also shown in Figure 2B. The main effect of cue type was significant, *F*(1, 28) = 41.70, *p* < 0.001, *η*^2^_*p*_ = 0.60. The main effect of probe stimulus type was significant, *F*(2, 56) = 4.10, *p* = 0.022, *η*^2^_*p*_ = 0.13. Additionally, the interaction between cue type and probe stimulus type was significant, *F*(2, 56) = 5.17, *p* = 0.009, *η*^2^_*p*_ = 0.16. Post hoc comparisons showed that when the probe stimulus was the target, response time under the neutral cue condition was significantly longer than under the valid cue condition [*t*(28) = 5.21, *p* < 0.001, Cohen’s *d* = 0.94]. When the probe stimulus was a non-target, response time under the neutral cue was also significantly longer than under the valid cue [*t*(28) = 5.38, *p* < 0.001, Cohen’s *d* = 0.99]. When the probe stimulus was a new object, response time under the neutral cue condition was again significantly longer than under the valid cue condition [*t*(28) = 9.00, *p* < 0.001, Cohen’s *d* = 1.68].

#### 2.2.2. Retro-Cue Benefit Index (RBI)

**Figure 3:**
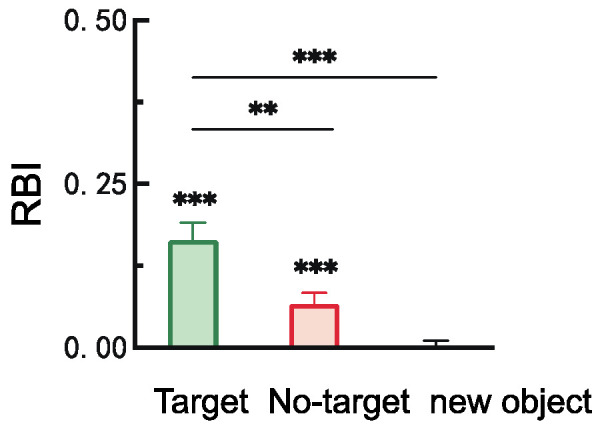
Retro-cue benefit indices for different probe stimulus types (** *p* < 0.01; *** *p* < 0.001).

The retro-cue benefit indices under the three probe stimulus conditions, along with the results of one-sample *t*-tests comparing each index to the baseline (0), are shown in Figure 3. The results indicated that the benefit indices for both the target and non-target items were significantly greater than baseline, *t*(28) = 5.90, *p* < 0.001, Cohen’s *d* = 1.10; *t*(28) = 3.70, *p* < 0.001, Cohen’s *d* = 0.69. Moreover, the benefit index for the target item was significantly greater than that for both the non-target and novel items, *t*(28) = 3.50, *p* = 0.003, Cohen’s *d* = 0.93; *t*(28) = 5.77, *p* < 0.001, Cohen’s *d* = 1.53.

#### 2.2.3. ERP Components

**Figure 4:**
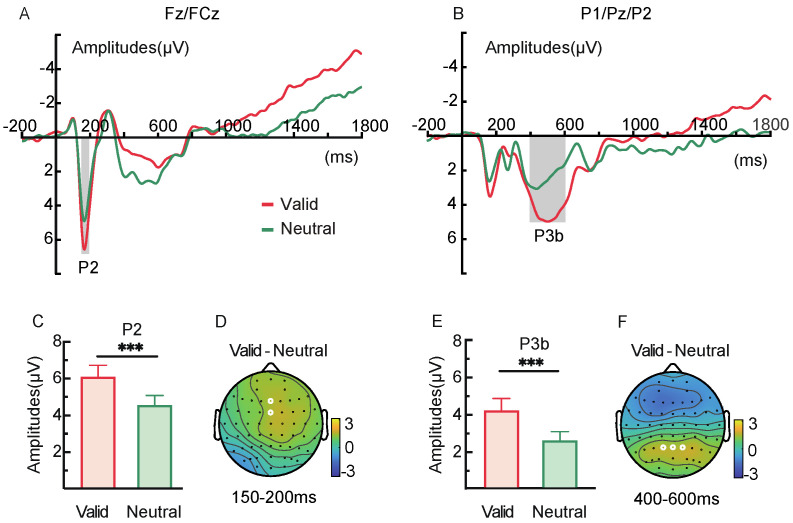
Average amplitudes of the P2 and P3b components (**A**,**B**) under valid and neutral retro-cue conditions in Experiment 1, along with topographic maps of the difference waves (**D**,**F**), and statistical comparisons of mean amplitudes (**C**,**E**).The white dots indicate the electrodes used for statistical analysis. (*** *p* < 0.001).

In the 150–200 ms window following the retro-cue presentation, the P2 component at frontal electrodes (Fz/FCz) (Figure 4A,D) showed more positive amplitudes under the valid retro-cue condition compared to the neutral retro-cue, *t*(28) = 4.42, *p* < 0.001, Cohen’s *d* = 0.48 (Figure 4C). Additionally, in the 400–600 ms window, the P3b component at parietal electrodes (P1/Pz/P2) (Figure 4B,F) was also more positive for valid retro-cues than for neutral retro-cues, *t*(28) = 4.12, *p* < 0.001, Cohen’s *d* = 0.62 (Figure 4E).

#### 2.2.4. CDA Component

**Figure 5:**
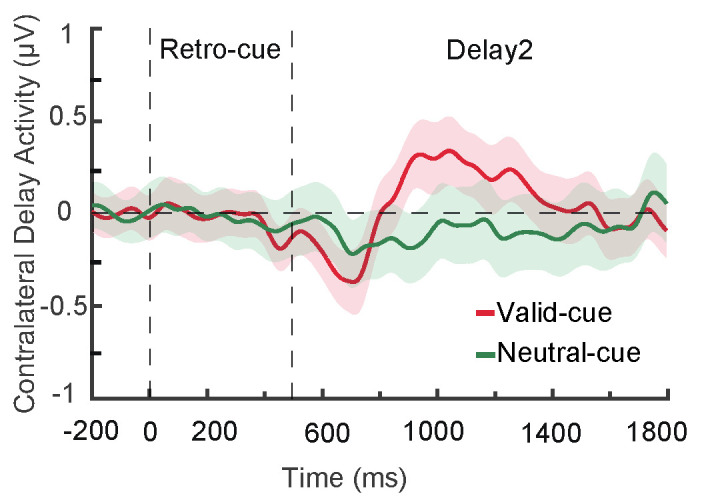
CDA components under valid and neutral retro-cue conditions.

During the retro-cue and delay phases, no significant CDA component was observed in either cue condition, and the difference in CDA amplitudes between valid and neutral cues was not significant (Figure 5).

#### 2.2.5. Time–Frequency Analysis

**Figure 6:**
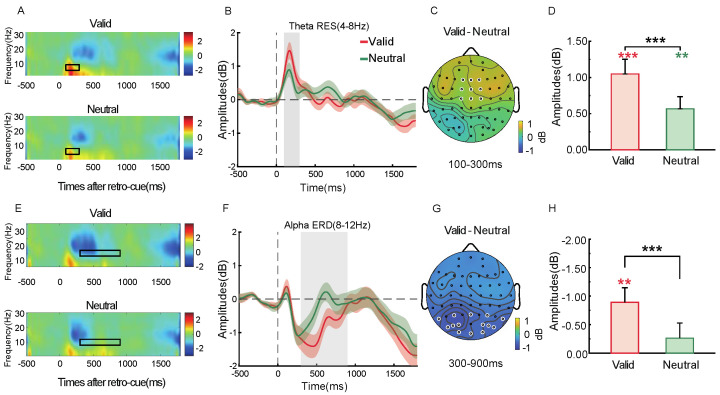
In the frontal region: time–frequency plots under valid and neutral retro-cue conditions (**A**), average theta power (4–8 Hz) (**B**), *t*-test results (**D**), and the corresponding topographical difference map for 100–300 ms (**C**). In the parieto-occipital region: time–frequency plots under valid and neutral conditions (**E**), average alpha power (8–12 Hz) (**F**), *t*-test results (**G**), and the corresponding topographical difference map for 300–900 ms (**H**). Black rectangles and gray shading indicate significant time intervals; white dots represent the electrodes used for the analysis. The white dots indicate the electrodes used for statistical analysis. Red asterisks indicate one-sample *t*-test results under the valid condition, and green asterisks indicate those under the neutral condition. (** *p* < 0.01; *** *p* < 0.001).

In the frontal region (F1/Fz/F2/FC1/FCz/FC2/C1/Cz/C2), theta synchronization appeared 100–300 ms after retro-cue onset (Figure 6A,B). As shown in Figure 6D, both valid and neutral retro-cue conditions showed significant theta synchronization [*t*(28) = 5.13, *p* < 0.001, Cohen’s *d* = 0.97; *t*(28) = 3.36, *p* = 0.002, Cohen’s *d* = 0.64], with stronger theta synchronization in the valid condition compared to the neutral one, *t*(28) = 4.07, *p* < 0.001, Cohen’s *d* = 0.76. The topographical distribution of theta power differences between conditions is shown in Figure 6C. Alpha desynchronization was observed in the parieto-occipital region (P3/4, PO3/4, PO5/6, P7/8, PO7/8, O1/2, Pz, Oz) 300–900 ms after retro-cue onset (Figure 6E,F). As shown in Figure 6H, significant alpha desynchronization was observed under the valid cue condition, *t*(28) = 3.48, *p* = 0.002, Cohen’s *d* = 0.66. Alpha desynchronization was also significantly stronger in the valid than the neutral cue condition, *t*(32) = 3.76, *p* < 0.001, Cohen’s *d* = 0.71. The corresponding topographical map of alpha power differences is shown in Figure 6G.

#### 2.2.6. Phase–Amplitude Coupling (PAC)

**Figure 7:**
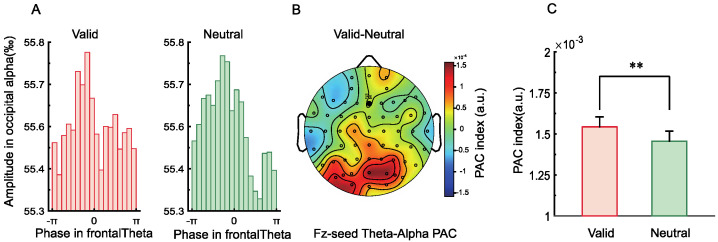
(**A**) Alpha amplitude at different theta phases under valid and neutral cue conditions. (**B**) Topographical difference map of theta–alpha phase–amplitude coupling (PAC) between Fz and all electrodes, comparing valid and neutral cue conditions. (**C**) PAC indices calculated between Fz and all parieto-occipital electrodes were significantly higher under the valid cue condition than under the neutral condition (** *p* < 0.01).

Since retro-cues may enhance target representations by modulating frontal theta phase and posterior alpha power, we examined long-range fronto-parietal PAC to test whether valid retro-cues strengthened this connectivity and thereby optimized WM performance. As shown in Figure 7A, alpha amplitudes across theta phase bins showed an uneven distribution in the valid cue condition, whereas the distribution was more uniform in the neutral condition. Figure 7B presents the topographical map of PAC differences between conditions for theta phase at Fz and alpha amplitude across all electrodes. We found significantly higher PAC between Fz and parieto-occipital electrodes in the valid cue condition compared to the neutral condition, *t*(28) = 3.36, *p* = 0.002, Cohen’s *d* = 0.63 (Figure 7C). These results suggest that frontoparietal connectivity may represent one important mechanism supporting retrospective attention during the WM retention phase.

#### 2.2.7. Decoding of Retro-Cues

**Figure 8:**
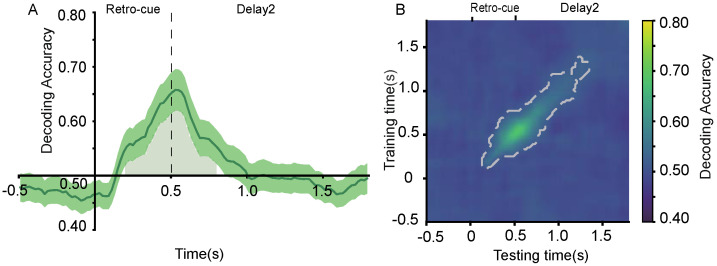
(**A**) Decoding of EEG signals for retro-cue types (valid vs. neutral cues). The shaded area beneath the curve (200–800 ms) indicates time points where decoding accuracy was significantly above chance level. (**B**) Temporal generalization matrix. The region enclosed by the gray contour indicates significant clusters based on permutation testing at the *p* < 0.05 level.

To determine whether EEG activity could distinguish between cue types (valid vs. neutral), we performed MVPA on the two retro-cue conditions. Results showed that decoding accuracy was significantly above chance between 200–800 ms (Figure 8A). Further time generalization analysis (Figure 8B) indicated that this neural activity pattern was sustained from 200 to 1600 ms.

### 2.3. Discussion

Under low-load conditions, compared to neutral retro-cues, valid retro-cues significantly enhanced recognition performance for both target and non-target items, demonstrating the advantage of retro-cues in facilitating the retrieval of relevant information [21,33]. In addition, valid retro-cues significantly reduced the response time to probe stimuli relative to neutral retro-cues, suggesting that valid retro-cues enhanced the perceptual processing speed of incoming stimuli, thereby facilitating comparison with items retained in visual working memory [42,43]. ERP results revealed that valid cues elicited increased amplitudes in the P2 and P3b components, reflecting stronger early attentional allocation and later cognitive updating processes [35,36]. Time–frequency analyses further showed that retro-cues induced significant frontal theta synchronization and parietal alpha desynchronization. Notably, PAC between frontal theta and parietal alpha activities was significantly enhanced under valid cue conditions, suggesting that functional coupling between frontal and posterior regions may jointly support the strengthening of target representations [44]. EEG decoding further confirmed that the optimal time window for retro-cue effects occurred around 500 ms [6], and that valid cues allowed for sustained differentiation of neural representations over a prolonged time window, indicating a lasting modulatory influence of the cue. In sum, the results of Experiment 1 suggest that under low working memory load, the formation of representational prioritization primarily relies on the enhancement of target information rather than suppression of non-target items.

## 3. Experiment 2

### 3.1. Method

#### 3.1.1. Participants

Using G*Power 3.19 [37], we calculated that a minimum of 28 participants would be required to detect a medium effect size (Cohen’s f = 0.25) with α = 0.05 and power (1 − β) ≈ 0.80. Considering possible data loss due to EEG artifacts and participant dropout, we recruited another 30 participants for Experiment 2. One participant was excluded due to excessive EEG artifacts (fewer than 30 valid trials). The final sample included 29 participants (10 males), aged 18–28 years. Other demographic and eligibility criteria were the same as in Experiment 1. Reaction time data exceeding ±3 standard deviations from the mean were excluded, accounting for approximately 1.4% of the total data.

#### 3.1.2. Procedure

**Figure 9:**
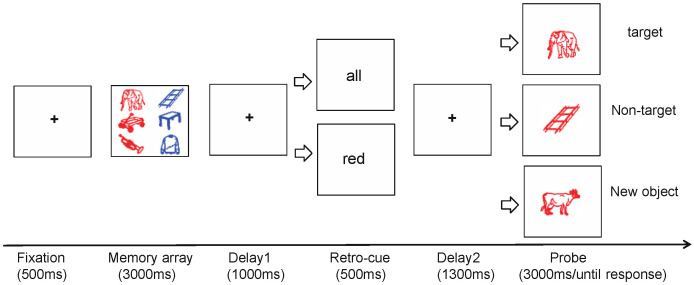
Experimental procedure of Experiment 2.

The procedure of Experiment 2 was largely like that of Experiment 1. The key difference was that the memory array contained six objects presented simultaneously, divided into two colors. The objects in each color group were displayed on opposite sides of the screen (see Figure 9).

### 3.2. Results

#### 3.2.1. Accuracy and Response Time

**Figure 10:**
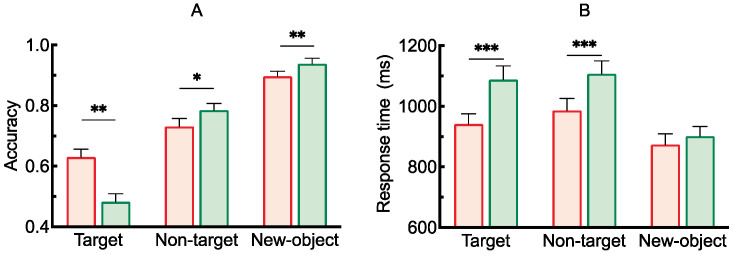
(**A**) Accuracy under different conditions, (**B**) Response time under different conditions (* *p* < 0.05; ** *p* < 0.01; *** *p* < 0.001).

The results of the analysis of variance on accuracy under different conditions are shown in Figure 10A. The main effect of cue type was not significant, *F*(1, 28) = 0.94, *p* = 0.341, *η*^2^_*p*_ = 0.65. The main effect of probe stimulus type was significant, *F*(2, 56) = 138.135, *p* < 0.001, *η*^2^_*p*_ = 0.83. Additionally, there was a significant interaction between cue type and probe stimulus type, *F*(2, 56) = 12.08, *p* < 0.001, *η*^2^_*p*_ = 0.30. Post hoc comparisons showed that when the probe stimulus was the target, accuracy under the valid cue condition was significantly higher than under the neutral cue condition [*t*(28) = 3.02, *p* = 0.006, Cohen’s *d* = 0.56]. When the probe stimulus was a non-target, accuracy under the neutral cue condition was significantly higher than under the valid cue condition [*t*(28) = 2.25, *p* = 0.031, Cohen’s *d* = 0.42]. When the probe stimulus was a new object, accuracy under the neutral cue condition was also significantly higher than under the valid cue condition [*t*(28) = 3.73, *p* = 0.001, Cohen’s *d* = 0.69].

The results of the analysis of variance on response time under different conditions are shown in Figure 10B. The main effect of cue type was significant, *F*(1, 28) = 50.40, *p* < 0.001, *η*^2^_*p*_ = 0.64. The main effect of probe stimulus type was also significant, *F*(2, 56) = 68.91, *p* < 0.001, *η*^2^_*p*_ = 0.71. In addition, the interaction between cue type and probe stimulus type was significant, *F*(2, 56) = 21.60, *p* < 0.001, *η*^2^_*p*_ = 0.44. Post hoc comparisons showed that when the probe stimulus was the target, response time under the neutral cue condition was significantly longer than under the valid cue condition [*t*(28) = 7.35, *p* < 0.001, Cohen’s *d* = 1.37]. When the probe stimulus was a non-target, response time under the neutral cue condition was also significantly longer than under the valid cue condition [*t*(28) = 7.06, *p* < 0.001, Cohen’s *d* = 1.21]. When the probe stimulus was a new object, the difference in response time between the two cue conditions was not significant [*t*(28) = 1.75, *p* = 0.092, Cohen’s *d* = 0.33].

#### 3.2.2. Retro-Cue Benefit Index (RBI)

**Figure 11:**
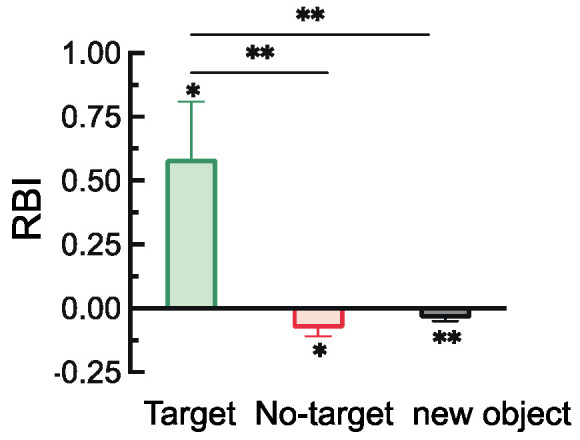
Retro-cue benefit indices for different probe stimulus types (* *p* < 0.05; ** *p* < 0.01).

As shown in Figure 11, one-sample *t*-tests comparing the retro-cue benefit indices of the three probe types to baseline (0) revealed the following: the benefit index for target items was significantly greater than zero, *t*(28) = 2.57, *p* = 0.016, Cohen’s *d* = 0.48; the indices for non-target and novel items were significantly below zero, *t*(28) = −2.38, *p* = 0.024, Cohen’s *d* = −0.44; *t*(28) = −3.08, *p* = 0.005, Cohen’s *d* = −0.57. In addition, the benefit index for target items was significantly greater than that for both non-target items, *t*(28) = 3.54, *p* = 0.002, Cohen’s *d* = 0.93, and novel items, *t*(28) = 3.33, *p* = 0.005, Cohen’s *d* = 0.87.

#### 3.2.3. ERP Components

**Figure 12:**
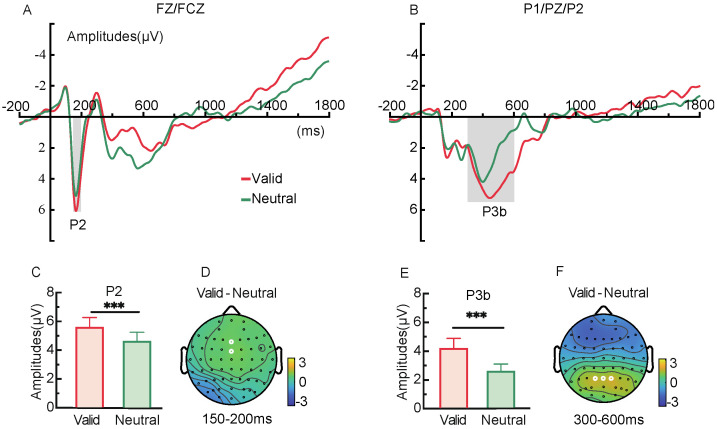
Mean amplitudes of the P2 and P3b components (**A**,**B**) under valid and neutral retro-cue conditions in Experiment 2, along with the corresponding difference wave topographies (**D**,**F**). Panels (**C**,**E**) display the average amplitude comparisons.The white dots indicate the electrodes used for statistical analysis. (*** *p* < 0.001).

Under the valid retro-cue condition, both the P2 and P3b components exhibited significantly more positive amplitudes compared to the neutral cue condition (see Figure 12), *t*(28) = 3.81, *p* < 0.001, Cohen’s *d* = 0.28; *t*(28) = 4.28, *p* < 0.001, Cohen’s *d* = 0.65.

#### 3.2.4. CDA Component

**Figure 13:**
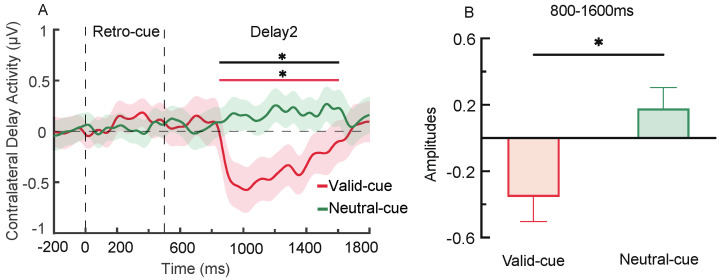
CDA components under different retro-cue conditions (**A**) and the mean amplitude during the 800–1600 ms interval (**B**). In panel A, the red line indicates a significant difference between the CDA amplitude and zero in the valid retro-cue condition, while the black line indicates a significant difference between the valid and neutral retro-cue conditions (* *p* < 0.05).

In the retro-cue and delay phases, the CDA amplitude in the valid cue condition was significantly lower than zero, *t*(28) = −2.39, *p* = 0.024, Cohen’s *d* = −0.20, and also significantly lower than that in the neutral cue condition (see Figure 8B), *t*(28) = −2.74, *p* = 0.011, Cohen’s *d* = −0.51, indicating the presence of a CDA component (Figure 13A,B).

#### 3.2.5. Time–Frequency Analysis

**Figure 14:**
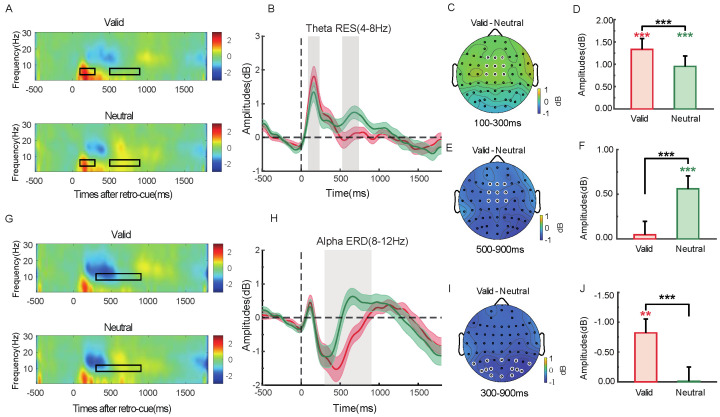
Time–frequency plots in the frontal region for valid and neutral cue conditions (**A**), average theta power (4–8 Hz) plots (**B**), *t*-test results for theta power during 100–300 ms and 500–900 ms (**D**,**F**), and corresponding topographical difference maps for these time windows (**C**,**E**). In the parieto-occipital region, time–frequency plots for valid and neutral cue conditions (**G**), average alpha power (8–12 Hz) plots (**H**), *t*-test results for alpha power (**J**), and the corresponding topographical difference map for the 300–900 ms time window (**I**). Black boxes and gray shaded areas indicate significant time periods; white dots represent electrodes used in the analysis.The white dots indicate the electrodes used for statistical analysis. Red asterisks indicate one-sample *t*-test results under the valid condition, and green asterisks indicate those under the neutral condition. (** *p* < 0.01; *** *p* < 0.001).

Theta (4–8 Hz) results showed a synchronization effect in the frontal region (F1/Fz/F2/FC1/FCz/FC2/C1/Cz/C2) during the 100–300 ms window following the retro-cue (see Figure 14A,B). As shown in Figure 12D, both valid and neutral retro-cues elicited significant theta synchronization [*t*(28) = 5.54, *p* < 0.001, Cohen’s *d* = 1.03; *t*(28) = 4.07, *p* < 0.001, Cohen’s *d* = 0.76]. Moreover, theta synchronization was significantly stronger in the valid cue condition compared to the neutral cue condition [*t*(28) = 4.45, *p* < 0.001, Cohen’s *d* = 0.83]. The corresponding topographical difference in theta power between conditions is shown in Figure 14C. During the 500–900 ms time window following the retro-cue (Figure 14A,B), theta synchronization also emerged. As shown in Figure 12F, significant theta synchronization was observed in the neutral cue condition [*t*(28) = 3.84, *p* < 0.001, Cohen’s *d* = 0.71], with stronger synchronization in the valid cue condition [*t*(28) = 3.87, *p* < 0.001, Cohen’s *d* = 0.72]. The topographical difference between valid and neutral conditions is shown in Figure 14E. Alpha (8–12 Hz) desynchronization was observed in the parieto-occipital region (P3/4, PO3/4, PO5/6, P7/8, PO7/8, O1/2, Pz, Oz) during the 300–900 ms window following the retro-cue (Figure 14G,H). As shown in Figure 14J, significant alpha desynchronization was found in the valid cue condition [*t*(28) = 3.54, *p* = 0.001, Cohen’s *d* = 0.66], and it was significantly stronger than that in the neutral cue condition [*t*(32) = 4.41, *p* < 0.001, Cohen’s *d* = 0.81]. The corresponding alpha power difference topography between the two cue conditions is shown in Figure 14I.

#### 3.2.6. Phase–Amplitude Coupling (PAC)

**Figure 15:**
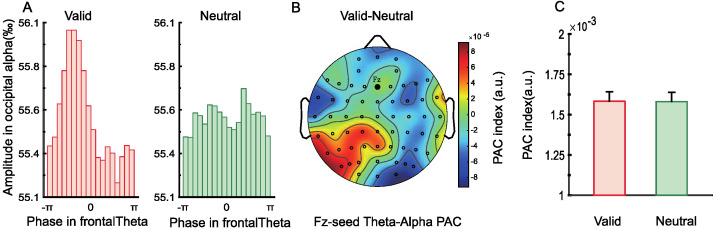
(**A**) Alpha amplitudes across different theta phase bins under valid and neutral cue conditions. (**B**) Topographical difference map of theta–alpha phase–amplitude coupling (PAC) between valid and neutral cue conditions, computed between Fz and all electrodes. (**C**) Results of the *t*-test comparing PAC indices between Fz and all parieto-occipital electrodes under valid versus neutral cue conditions.

The alpha amplitude at different theta phase bins (Figure 15A) showed a non-uniform distribution under the valid retro-cue condition, while the distribution was relatively uniform under the neutral cue condition. Figure 15B illustrates the topographical difference map of theta−alpha PAC between the Fz electrode and all other electrodes under valid versus neutral conditions. However, no significant difference in theta–alpha PAC was found between the Fz electrode and parieto-occipital electrodes across the two cue conditions.

#### 3.2.7. Decoding of Retro-Cues

**Figure 16:**
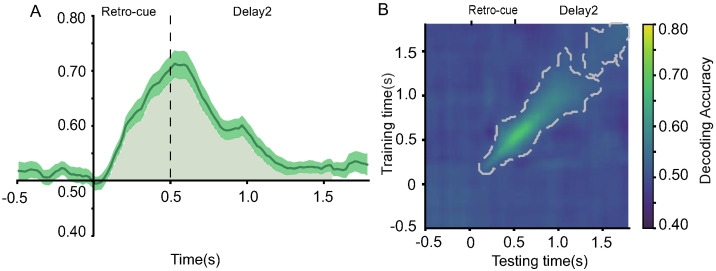
EEG decoding of retro-cue types (valid vs. neutral). (**A**) The shaded area under the curve (200–800 ms) indicates that decoding accuracy was significantly above chance level. (**B**) The region enclosed by the thick gray line represents significant clusters identified via permutation testing at the *p* < 0.05 level.

The MVPA decoding analysis of the two types of retro-cues revealed that decoding accuracy was significantly above chance level between 100 and 1560 ms (see Figure 16A). Furthermore, the temporal generalization analysis (Figure 16B) indicated that this neural activity pattern could be sustained from 100 to 1800 ms.

### 3.3. Discussion

Experiment 2 further investigated the mechanisms underlying the formation of representational prioritization in VWM under high memory load conditions. Behavioral results indicated that valid retro-cues not only enhanced the recognition accuracy of target items but also reduced the recognition rates for non-target and novel items. This suggests that when cognitive resources are limited, retro-cues facilitate prioritization through both target enhancement and non-target suppression. Moreover, valid retro-cues continued to reduce response times to probe stimuli even under high load [5]. ERP results showed that valid cues elicited significantly greater P2 and P3b amplitudes compared to neutral cues, reflecting stronger attentional allocation and memory updating processes [35,36]. Furthermore, during the maintenance phase (800–1600 ms), significant CDA components were observed under valid retro-cues conditions, indicating the deletion of non-targets [7]. Time–frequency analysis revealed that valid retro-cues induced stronger frontal theta synchronization within 100–300 ms, indicating that target information was quickly selected and received attentional support. However, this effect weakened during the 500–900 ms interval, while theta synchronization under neutral cues continued to increase, implying that sustained control was required for neutral cues [18,20]. In the parieto-occipital region, alpha desynchronization was observed between 300–900 ms, with stronger effects under valid cues, consistent with the role of alpha oscillations in supporting target processing and feature integration [25,26]. PAC analysis revealed no significant enhancement in frontoparietal coupling, suggesting that under high load, cue-related modulation may rely more on localized neural activity rather than long-range connectivity [44]. Meanwhile, MVPA decoding demonstrated that valid and neutral cues could be reliably distinguished throughout the 100–1800 ms window, indicating a sustained modulatory effect of retro-cues on memory representations [8]. In summary, under high memory load, the formation of representational prioritization depends on the synergistic operation of target enhancement and non-target suppression. This prioritization process appears to be supported by localized theta and alpha oscillatory activity.

## 4. General Discussion

This study employed a retro-cue paradigm to investigate how working memory (WM) load modulates the mechanisms underlying the formation of representational prioritization and their associated neural oscillatory patterns. The results showed that under low load conditions (Experiment 1), valid retro-cues significantly facilitated the retrieval of both target and non-target items. The amplitudes of the P2 and P3b components under valid retro-cue conditions were significantly larger than those under neutral cue conditions, and no significant CDA component was observed. Time–frequency analysis revealed that valid retro-cues induced frontal theta synchronization and posterior alpha desynchronization, along with enhanced PAC between frontal theta and parietal alpha activities, reflecting the activation and integration of target information. Under high load conditions (Experiment 2), valid retro-cues facilitated only the retrieval of target items while suppressing non-target items. The amplitudes of the P2 and P3b components were again significantly larger under valid retro-cue conditions than under neutral cue conditions, and a significant CDA component emerged in the valid retro-cue condition. Compared to the low-load condition, the duration of theta synchronization in the valid retro-cue condition was shorter under high load, and the PAC between frontal theta and parietal alpha activities did not differ significantly between valid and neutral cues. In sum, the regulatory mechanisms by which valid retro-cues modulate behavioral and neural responses differ depending on WM load, suggesting that the formation of representational prioritization is dynamically influenced by the availability of cognitive resources.

### 4.1. The Generality of the Target Enhancement Mechanism

Previous studies have suggested that target enhancement is not influenced by the validity of retro-cues [7], the type of cue [45], or memory load [33]. In retro-cue tasks, internal attention is compulsorily directed toward the cued item, enabling the target to automatically receive more cognitive resources. This enhancement is based on the reactivation of existing memory representations and reflects the prioritized allocation of resources [46]. In the present study, target enhancement was observed under both low- and high-load conditions. Under low load (Experiment 1), resources were relatively sufficient, and the resources allocated to targets did not compromise those available for non-targets; thus, no deletion of non-targets occurred. In contrast, under high load (Experiment 2), the resources available for enhancing targets were limited and had to be drawn from the resources that would otherwise support non-targets. These findings further support the notion that target enhancement is a robust and adaptive mechanism across various task conditions and serves as a primary mechanism for prioritization in visual working memory [7,31,33,34].

ERP results showed that the amplitudes of both the P2 and P3b components were more positive in the valid retro-cue condition compared to the neutral retro-cue condition. In retro-cue paradigms, an increased P2 amplitude suggests that the memory contents indicated by the valid retro-cue received enhanced attentional resources [35]. The increased parietal P3b amplitude reflects that the cued memory contents were updated accordingly [36]. These findings support the idea that the formation of representational prioritization is realized through internal attentional selection and updating [47]. Further MVPA decoding analysis demonstrated that, following the onset of the retro-cue and throughout the delay period, decoding accuracy between valid and neutral cues was significantly above chance under both low and high memory load conditions. This indicates distinct neural patterns underlying the processing of valid and neutral retro-cues in both conditions. Notably, the decoding difference between the two cue types peaked around 500 ms, aligning with the typical time course of item-based retro-cue effects reported in previous studies [8]. Oscillatory evidence further supported this mechanism. Stronger frontal theta synchronization (100–300 ms) was observed following valid retro-cues under both load conditions, indicating rapid top-down modulation from the prefrontal cortex toward the target representations [18,22]. Simultaneously, alpha desynchronization in the parieto-occipital region was observed in both load conditions following valid retro-cues. The reduction in alpha power suggests that the parietal–occipital network may facilitate the maintenance and retrieval of target information by “opening sensory channels” and allocating greater processing resources [48,49]. In addition, temporal generalization analysis of the decoding results confirmed the stability of internal attentional selection and updating processes. Temporal generalization assesses whether neural activity patterns at one time point generalize to another, reflecting whether these time points involve different stages of the same cognitive process. It serves as an important index of the temporal stability of mental representations [50]. Thus, the selection and updating of memory content triggered by the retro-cue represent a continuous and stable process.

### 4.2. Resource Conditions for the Occurrence of Non-Target Suppression Mechanisms

Previous studies investigating the formation of representational prioritization typically ensured 100% cue validity in retro-cue paradigms, which made it difficult to simultaneously measure participants’ behavioral responses to both targets and non-targets [19,51,52]. In this study, we combined the valid cue color with non-target objects, thus balancing cue validity and the measurement of responses to non-targets. This approach allowed us to accurately capture behavioral and neural evidence of non-target suppression. When retro-cues triggered the deletion of non-targets from the maintenance stage, participants’ recognition performance for non-targets sharing the same color decreased.

Under low memory load (Experiment 1), valid retro-cues facilitated the recognition of both target and non-target items, indicating that target enhancement occurred following cue presentation, while non-target suppression did not. ERP results showed increased P2 and P3b amplitudes under valid cue conditions but no significant CDA component, further suggesting that attention resources were preferentially allocated to the target, whereas non-targets remained in working memory [7,35,36]. In contrast, under high memory load (Experiment 2), valid retro-cues not only improved recognition of targets but also impaired recognition of non-targets, and this effect was accompanied by a significant CDA component, indicating that non-target information had been removed from working memory [7]. These results suggest that working memory load directly influences the mechanisms of representational prioritization. When cognitive resources are sufficient under low load, both target and non-target items can receive ample resources, and non-targets are not excluded. During non-target retrieval, the target indicated by the cue may become an attended yet task-irrelevant item. In such cases, the brain may actively suppress the target information through a “reselection” mechanism to reduce or eliminate its interference with other content [53]. This reselection process can even enhance non-target retrieval, resulting in a non-target benefit. In contrast, under high memory load, available resources are insufficient. Enhancing the representation of the target via a retro-cue comes at the cost of reducing resources allocated to non-targets [33,54], leading to the suppression or removal of non-targets to ensure the stability of target representation [55,56].

Notably, this finding contrasts with research in the domain of external attention, where “high load impairs suppression” has been consistently reported. For example, Wei et al. (2020) found that under increased working memory load, individuals’ ability to suppress irrelevant external stimuli during visual search tasks significantly declined [57]. This discrepancy suggests fundamental differences in the resource regulation mechanisms between internal and external attention. External attention primarily depends on continuous environmental monitoring and suppression of external input. When resources are limited, executive control mechanisms are impaired, leading to weakened suppression [58,59]. In contrast, internal attention targets information already encoded in working memory. Its suppression process is more akin to “active deletion” or selective removal and is often triggered under conditions of resource scarcity [6,12]. Therefore, while high load weakens suppression in external attention, it activates suppression in internal attention, reflecting adaptive differences in how the two attentional systems respond to resource bottlenecks [6,60].

### 4.3. Modulatory Effects of Visual Working Memory Load on Neural Oscillations in Representational Prioritization

Neural oscillations play a central regulatory role in the formation of representational prioritization in working memory. Frontal theta synchronization, posterior alpha desynchronization, and PAC between these two rhythms are widely considered critical mechanisms supporting internal attention and information control [22,24,44]. The present study further demonstrated that these oscillatory indices exhibit distinct patterns under varying working memory load, suggesting that the availability of cognitive resources directly modulates how brain rhythms participate in the prioritization process.

Within 100–300 ms after the presentation of the retro-cue, significant frontal theta synchronization was observed under both load conditions, with stronger synchronization for valid retro-cues than neutral cues. This finding supports the fundamental role of frontal theta rhythms in initiating target selection, potentially by rhythmically coordinating the allocation of internal attention [22,23]. However, theta activity in the 500–900 ms interval showed sensitivity to load: under low load, theta synchronization rapidly diminished, indicating that brief activation was sufficient for target representation; under high load, theta synchronization induced by valid retro-cues declined quickly, while theta activity under neutral cue conditions remained elevated. This suggests that in resource-limited contexts, the brain adopts an energy-saving “fast initiation–rapid withdrawal” strategy, whereas in the absence of clear guidance (i.e., neutral cue), sustained theta is needed to maintain the activation of multiple memory representations [6,17,20]. These results imply that theta rhythms not only encode target initiation but also support dynamic regulation of resource allocation. Alpha oscillations have been widely associated with the gating of sensory input and the suppression or release of perceptual channels [24,49]. In this study, during the 300~900 ms interval following retro-cue presentation, significant alpha desynchronization was observed in the parieto-occipital regions under both load conditions, with a greater decrease in power for valid compared to neutral retro-cues. This pattern suggests that alpha desynchronization consistently supports target enhancement, reflecting the opening of sensory processing channels and increased transmission efficiency for prioritized information [25,61]. Although previous research has indicated that alpha desynchronization may also reflect the suppression of non-targets [24], the consistent pattern across both load conditions in this study suggests that it primarily reflects a general mechanism of target enhancement rather than a load-dependent suppression of irrelevant items. Cross-frequency PAC between frontal theta and posterior alpha is thought to reflect interregional coordination between frontal and posterior brain areas, facilitating attentional regulation and information integration [44]. In the low-load condition, valid retro-cues significantly enhanced theta–alpha PAC, indicating that when resources are ample, the brain tends to engage in interregional frequency coupling to optimize target representation and attentional guidance [62]. In contrast, under high-load conditions, no significant PAC differences were observed between valid and neutral cues, possibly reflecting a shift toward local processing strategies in the face of constrained cognitive resources [18].

Taken together, these findings reveal that working memory load modulates neural rhythmic mechanisms of prioritization. Under low-load conditions, the brain facilitates target processing through stable and sustained theta–alpha oscillations and their cross-frequency coupling. Under high-load conditions, however, target enhancement relies on shorter-duration theta activation and reduced cross-regional PAC, reflecting the brain’s flexibility and adaptability in dynamically allocating resources. These results provide a crucial temporal perspective on the neural underpinnings of representational prioritization in visual working memory.

### 4.4. Limitations and Future Directions

This study revealed how target enhancement and distractor suppression jointly shape representational priority in VWM under varying memory load. However, several limitations remain. First, the binary load manipulation (two vs. six items) may oversimplify the resource allocation process, which is likely continuous rather than discrete. Future studies should incorporate intermediate load levels to better capture the gradient nature of resource distribution in VWM [52]. Second, although the design ensured 100% retro-cue validity and included non-target probes, it also introduced possible response biases. Specifically, the yes/no response ratio was unbalanced, and non-target probes were re-colored versions of memory items, which may have affected participants’ expectations. Future studies should consider more balanced response formats and novel lures to reduce potential response-related confounds. Third, EEG analyses in the present study were limited to the cue and retention interval (Delay 2) and did not extend into the probe phase. This decision was made to avoid confounding effects introduced by the probe, which may act as a new external attentional cue and elicit additional perceptual and motor-related activity. Including the probe period would complicate the isolation of internal prioritization mechanisms. Nevertheless, future research could benefit from incorporating probe-locked EEG analyses using task designs optimized to dissociate probe-driven responses from internal attention dynamics.

## 5. Conclusions

This study employed a retro-cue paradigm to investigate how working memory load modulates the formation of representational prioritization and its neural oscillatory mechanisms. The results showed that target enhancement remained stable across load conditions, whereas non-target suppression emerged mainly under high load. Frontal theta and parietal–occipital alpha oscillations jointly supported target processing, with their cross-frequency coupling shaped by resource availability. These findings reveal how internal attention dynamically adapts to changing resource states in working memory.

## Data Availability

The data that support the findings of this study are available on request from the corresponding author. The data are not publicly available due to privacy or ethical restrictions.
